# Liver resection for ovarian cancer liver metastases as part of cytoreductive surgery is safe and may bring survival benefit

**DOI:** 10.1186/s12957-015-0652-0

**Published:** 2015-08-05

**Authors:** Nicolae Bacalbasa, Simona Dima, Vladislav Brasoveanu, Leonard David, Irina Balescu, Raluca Purnichescu-Purtan, Irinel Popescu

**Affiliations:** “Carol Davila” University of Medicine and Pharmacy, Bulevardul Eroii Sanitari 8, Bucharest, 050474 Romania; “Dan Setlacec” Center of General Surgery and Liver Transplantation, Fundeni Clinical Institute, Soseaua Fundeni 258, Bucharest, 022328 Romania; “Ponderas” Hospital, Bucharest, Romania; Department of Mathematical Methods and Models, Faculty of Applied Sciences, Politehnica University of Bucharest, Splaiul Independentei nr. 313, RO-060042 Bucharest, Romania

## Abstract

**Background:**

The aim of this study was to evaluate whether hepatic resections of ovarian cancer liver metastases provide a benefit in terms of survival as part of primary, secondary, tertiary, and even quaternary cytoreductive surgery.

**Methods:**

Data of patients submitted to surgery for ovarian cancer liver metastases at Fundeni Clinical Institute between January 2002 and April 2014 were retrospectively reviewed. Liver lesions were classified according to their origin in parenchymal and peritoneal lesions.

**Results:**

A total of 31 patients were identified: 11 of them underwent liver resection as part of primary cytoreduction, 15 at secondary cytoreduction, 3 at tertiary cytoreduction, and 2 at the time of quaternary cytoreduction. The survival of patients with primary cytoreduction including liver resection was significantly higher compared with that of patients with secondary cytoreductive surgery including liver resection (15.63 versus 6.63 months, log-rank *p* = 0.057, 90 % CI). The median survival of patients with hepatectomy for liver metastases from peritoneal seeding was higher than that of patients with hepatectomy for liver metastases from hematogenous origin (16.08 versus 12.66 months, log-rank *p* = 0.523).

**Conclusions:**

Hepatectomy in ovarian cancer liver metastases is a safe and effective procedure; however, a benefit in terms of survival in favor of peritoneal seeding has been systematically observed.

## Background

Ovarian cancer is the leading cause of death from gynecologic malignancies, with an estimated incidence of 22,240 new patients and 14,030 deaths due to the disease in the United States in 2013. As no effective screening tests exist and symptoms are discrete, most patients are diagnosed in advanced stages (III and IV) [1]. The mainstay of treatment is surgery, followed by platinum-based chemotherapy. As far as surgery is concerned, the idea of maximal cytoreduction is a clearly established concept with intense correlation between the amount of remaining tumoral tissue and survival [2, 3]. However, the majority of patients will recur at some point [4], and evidence is becoming available regarding the benefit of maximal cytoreduction in a secondary and even tertiary setting [5]. Also, the benefit of extensive upper abdominal procedures in the attempt to achieve complete cytoreduction is advocated [6]. Since up to 50 % of patients who die of ovarian cancer have liver involvement at autopsy [7], and some are up front diagnosed with liver metastases, one could question a possible benefit for liver resection especially for a disease in which the mainstay of treatment is maximal cytoreductive surgery. However, even though literature regarding the benefit of liver resection for non-colorectal non-neuroendocrine liver metastasis is increasingly being published, there are few studies regarding liver metastases from gynecologic primaries [7–18].

## Methods

Data of patients who underwent surgery for advanced and recurrent ovarian cancer at Fundeni Clinical Hospital between January 2002 and April 2014 were retrospectively reviewed. Written informed consent was obtained from the patients for the publication of these data. Patients submitted to liver resection for ovarian cancer liver metastases at the moment of primary, secondary, tertiary, and quaternary cytoreduction were considered eligible for the study. A total of 31 patients were identified: 11 of them underwent liver resection as part of primary cytoreduction, 15 of secondary cytoreduction, 3 of tertiary cytoreduction, and 2 at the moment of quaternary cytoreduction. Liver lesions were classified according to their origin in parenchymal and peritoneal lesions. Parenchymal lesions were defined as metastases with hematogenous origin, entirely developed intra-parenchymatously and entirely surrounded by liver parenchyma. Metastases originating from peritoneal seeding were considered those lesions developed on the liver surface with parenchymal invasion of at least 2 cm.

Preoperative, intraoperative, and postoperative data were collected retrospectively. The information included age at initial diagnosis; stage (Federation of Gynecology and Obstetrics (FIGO) systems); differentiation grade (well (G1), moderately (G2), or poorly (G3) differentiated carcinomas); histopathological type of the primary tumor; neoadjuvant chemotherapy; associated resections at the moment of each cytoreductive surgery; disease-free survival; number, maximum dimensions, type (peritoneal versus hematogenous), and intrahepatic distribution of the liver metastases; postoperative complications according to Dindo-Clavien classification; and overall survival estimated from the moment of performing hepatic surgery. Liver resection was considered major if more than two liver segments were resected, while minor hepatectomies referred to less extended resections. Type of resection was defined as follows: R0—no remnant tumoral tissue, R1—tumoral tissue between 0 and 1 cm, and R2—tumoral tissue >2 cm. Dates of death were obtained from the National Register of Population. The differences between different subgroups were analyzed by the log-rank test and considered significant if *p* < 0.05. Kaplan-Meyer survival curves were also used. Statistics and graphics were performed using the SPSS software version 17.0 (SPSS, Chicago, IL, USA).

## Results

From January 2002 to April 2014, 31 patients with liver resection for ovarian cancer liver metastases were evaluated.

### Primary cytoreduction for ovarian cancer including liver resection

At the moment of primary cytoreduction, 11 patients with mean age of 54 years (range, 24–70) old underwent liver resections for hepatic metastases. Two patients received neoadjuvant therapy. The main characteristics at the moment of primary cytoreduction are shown in Table 1. All 11 patients were submitted to surgery with radical intent. Table 2 shows the main perioperative characteristics of the patients included with stage IIIC–IV of disease. No operative 30-day postoperative deaths occurred, and the morbidity rate related to primary cytoreduction was 25 %. Severe postoperative complications (> grade III Dindo-Clavien) were present in two patients (25 %) with stage IV of disease.

**Table 1:** Characteristics of patients undergoing liver resection as part of primary cytoreductive surgery

Characteristics	No. of patients (*n* = 11)
Mean age	54 years (24–70)
FIGO stage	
IIC	1
IIIC	2
IV	8
Neoadjuvant chemotherapy	2
Histological type	
- Serous	8
- Endometrial	1
- Mucinous	–
- Other types	2
Differentiation grade	
- G1	2
- G2	5
- G3	4

*FIGO* Federation of Gynecology and Obstetrics systems

**Table 2:** Intraoperative findings, types of resection, and early postoperative outcomes of patients undergoing liver resection as part of primary cytoreductive surgery

Characteristics	No. of patients (*n* = 2)	No. of patients (*n* = 8)
	Stage IIIC	Stage IV
Number of liver metastasis		
- Single	2	7
- Multiple	–	1
Maximum diameter of liver lesions	2.33 cm (range, 2–3)	3.5 cm (range, 2–5)
Type of lesions		
- Peritoneal	2	1
- Parenchymatous	–	7
Distribution		
- Unilobular	2	7
- Bilobular	–	1
Type of resection		
- R0	2	6
- R1	–	–
- R2	–	1
- Palliation/biopsy	–	1
Type of liver resection		
- Minor hepatectomies	2	5
- Major hepatectomies (>2 segments)	–	1
- Radiofrequency ablation	–	1
Associated visceral resections		
- Total hysterectomy with bilateral adnexectomy	2	6
- Omentectomy	1	8
- peritonectomy	1	8
- Splenectomy	–	1
- Bowel resection	1	3
- Distal pancreatectomy	–	1
- Subtotal gastrectomy	–	1
- Diaphragmatic resection	1	2

Median survival of this group of patients was 15.63 months (range, 1–139), 34.33 months (range, 8–139) in patients with peritoneal liver metastases, and 15.63 months (range, 7–128) for patients with hematogenous liver metastases origin (log-rank *p* = 0.702).

### Secondary cytoreduction for ovarian cancer including liver resection

At the moment of secondary cytoreduction, 15 patients underwent liver resections. Disease-free survival (DFS) after primary cytoreductive surgery was 30 months (range, 7–88 months). The mean age at the moment of liver resection was 53 years (range, 33–71). The main characteristics of patients diagnosed with liver metastases at the moment of secondary cytoreduction are shown in Table 3. Six patients presented liver metastases with peritoneal origin, while the other nine patients were diagnosed with parenchymal lesions. The mean of the maximum diameter of liver lesions was 3.3 cm (range, 2–10 cm). An R0 resection was attempted in all patients; however, this was possible in 80 % of patients (12 of 15 patients). In two patients, an R2 resection was performed. The intraoperative findings and types of resection are summarized in Table 4. No postoperative death was recorded. Four of the 15 patients (27 %) encountered postoperative complications. Only two patients (13 %) developed complications related to liver resection—biliary fistula in one patient and hepatic abscess as well in one patient. The other complications were rather associated to the other synchronous surgical procedures, such as resection of pelvic recurrences involving bowel resection. Median survival for patients with liver resection at secondary cytoreduction for ovarian cancer was 6.16 months (range, 1–66) for patients with hematogenous liver metastases (*n* = 9) and 14.51 months (range, 4–138) for patients with liver metastases of peritoneal origin (*n* = 6) (log-rank *p* = 0.197).

**Table 3:** The main preoperative characteristics of patients undergoing liver resections as part of secondary cytoreductive surgery

Characteristics	No. of patients
Mean age at primary cytoreduction	51 years (32–68)
Mean age at secondary cytoreduction	53 years (33–71)
FIGO stage	
- IC	2
- IIC	1
- IIIB	2
- IIIC	10
Adjuvant chemotherapy	15
Histological type	
- Serous	15
- Endometrioid	–
- Mucinous	–
- Other types	–
Tumor grade	
- G1	4
- G2	5
- G3	6
DFS (months)	30 (7–88)

**Table 4:** Intraoperative findings and types of resection of patients undergoing liver resections as part of secondary cytoreductive surgery

Number of liver metastasis	
- Single	7
- Multiple	8
Maximum diameter of liver lesions	3.33 cm (range, 2–10)
Type of lesions	
- Peritoneal	6
- Parenchymatous	9
Distribution	
- Unilobular	13
- Bilobular	2
Type of resection	
- R0	12
- R1	–
- R2	2
- Palliation/biopsy	1
Type of liver resection	
- Minor hepatectomies	14
- Major hepatectomies (>2 segments)	1
Associated visceral resections	
- Cholecystectomy	4
- Splenectomy	4
- Bowel resection	4
- Cystectomy	3
- Subtotal gastrectomy	1
- Diaphragmatic resection	3

### Tertiary cytoreduction for ovarian cancer including liver resection

At the moment of tertiary cytoreduction, three patients were diagnosed with liver metastases. The mean age at the moment of tertiary cytoreduction was 60 years (range, 54–72), and the initial FIGO stages were I, II, and IIIC, respectively. The mean interval from the moment of initial diagnosis was 54 months (range, 14–91 months). Two of the three patients were diagnosed with unique hepatic metastases, both patients originating from initially early stage tumors, while the third patient, initially classified as IIIC, presented disseminated liver metastases with peritoneal origin. In the other two patients, the hematogenous route was incriminated. One patient was submitted to a major hepatic resection involving more than two segments for a 4-cm parenchymal lesion, while the other two were submitted to minor hepatectomies. The mean diameter of the tumors was 2.3 cm (range, 1–4 cm) while the histology revealed serous histology in all patients. One of the three patients experienced early re-operation for peritonitis due to urinary fistula after segmental ureteral resection and reimplantation; death occurred 6 days after re-operation. The other two patients experienced a survival of 63 and 70 months, the last one being still alive by the end of the study.

### Quaternary cytoreduction for ovarian cancer including liver resection

Two patients were submitted to liver resections during quaternary cytoreduction. Both patients were initially diagnosed with stage IIC ovarian cancer and experienced liver recurrence 3 years after initial surgery. The ages at the moment of liver resection were 39 and 48 years, respectively. Liver resections were performed at 40 and 33 months respectively from ovarian cancer diagnosis. One patient was diagnosed with hepatic recurrence with peritoneal origin while the second one was diagnosed with both parenchymal and peritoneal lesions. The liver lesion diameters were 2 and 4 cm, respectively; both patients were submitted to atypical hepatectomies; an R0 resection was feasible in a single patient, while in the other, an R1 resection was performed. Histopathological findings revealed a serous subtype in both patients. None of the patients experienced postoperative complications. The postoperative survival was 16 and 20 months after liver resection, both patients being dead of disease by the end of the study.

### Analysis of survival following hepatic resection based on the type of cytoreductive surgery, primary tumor grade, and stage

**Fig. 1:**
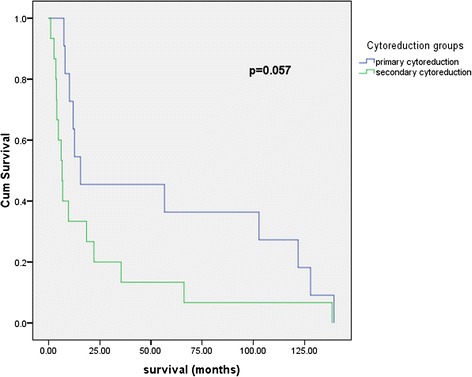
Survival following liver resection as part of primary cytoreductive surgery versus liver resections as part of secondary cytoreduction

The survival of patients with primary cytoreduction including liver resection was significantly higher compared with those of patients with secondary cytoreductive surgery including liver resection (15.63 versus 6.63 months, log-rank *p* = 0.057, 90 % CI) (Fig. 1). Survival of patients undergoing liver resection as part of primary cytoreduction was not statistically significant compared to the patients with liver resection as part of all other non-primary cytoreductive surgery (combining secondary, tertiary, and quaternary (log-rank *p* = 0.154)). Stage of the primary tumor was not significantly associated with survival; patients with primary tumor stages I–II had better median survival than those with stages III–IV, but the difference is not statistically significant (20.13 versus 12.35, log-rank *p* = 0.462). Survival was not significantly better for patients with G1–G2 grade of the primary tumor compared to G3 grade (0 = 0.092).

## Discussion

The important role of surgery in the treatment of ovarian cancer has been first advocated by Meigs [19], almost 100 years ago, later objectified by the landmark papers of Griffiths in 1970 [3]; currently, the inverse relationship between residual time following cytoreduction and survival is an undisputed fact [2]. Since the liver is either involved from the beginning or presents metachronous metastases following primary cytoreduction followed by adjuvant chemotherapy, a question may be raised about a possible benefit of liver resection as part of R0 cytoreduction either in a primary or subsequent setting. Due to the development of the surgical technique and intraoperative and postoperative intensive care, liver resection is becoming an increasingly safer procedure [20]; however, due to the success of hepatectomies for colorectal and neuroendocrine liver metastases, the role of liver resection for metastases originating from other primaries has increasingly been questioned.

**Fig. 2:**
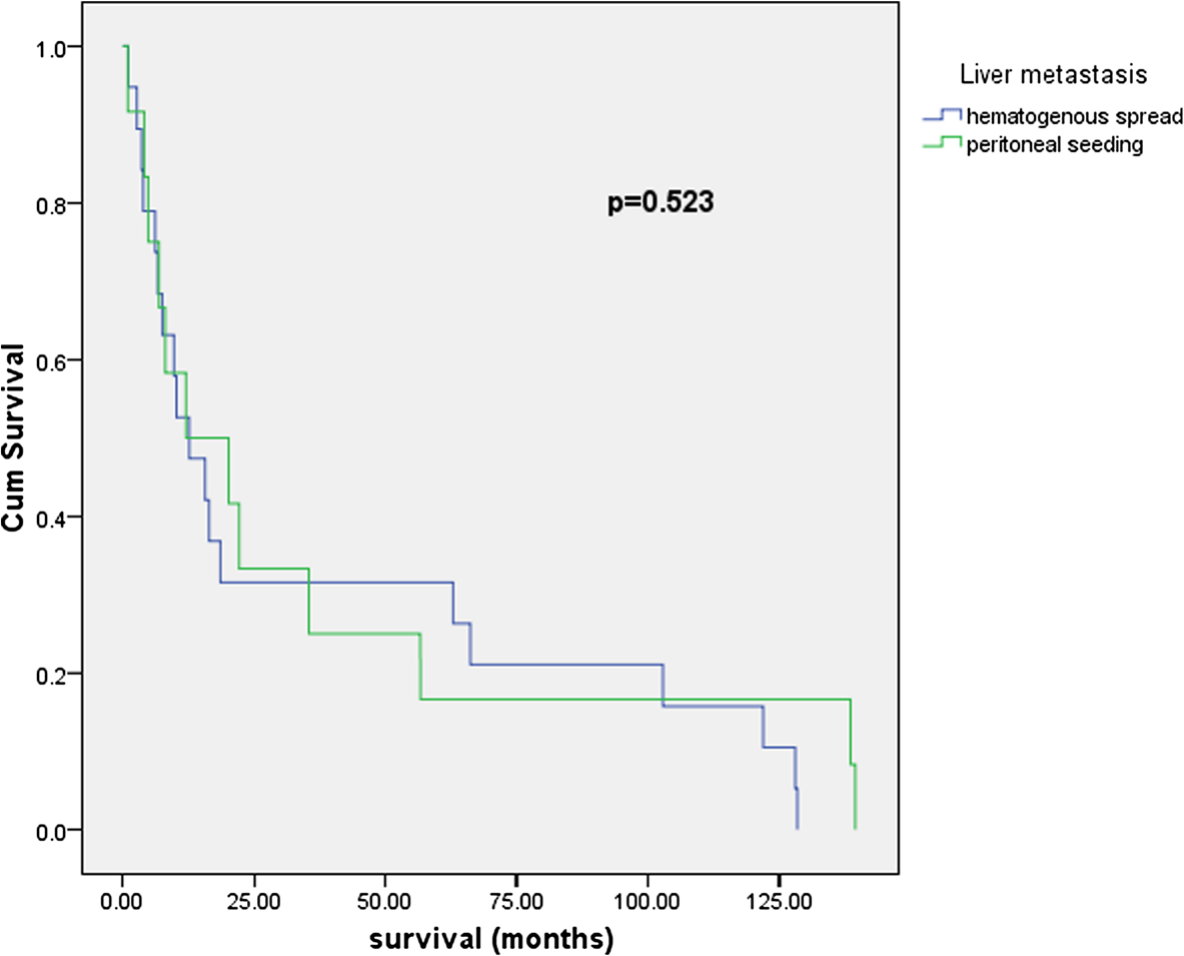
Survival following hepatectomy for liver metastases from peritoneal seeding versus hepatectomy for liver metastases from hematogenous origin. *Hematogeneous* hematogenous liver metastases, *Peritoneal* liver metastases originating from peritoneal seeding

Liver resection for metastases originating from gynecologic malignancies appears to be an extremely safe procedure since published papers on the subject report 0 mortality with morbidity rates ranging up to 37 % 8–17, which is impressive given the fact that in all studies, liver resection is accompanied by other significant debulking procedures in the upper and lower abdomen. Liver resection seems to bring survival benefit for patients with ovarian cancer liver metastases; Kamel et al. matched the patients who underwent liver surgery to patients similar in liver tumoral burden who underwent biopsy only, obtaining a clear difference with statistical significance of 53 versus 21 months median overall survival from time to diagnosis of liver metastases [9]. There also seems to be a clear survival difference following hepatectomy regarding the mechanism of liver involvement (hematogenous versus peritoneal seeding) [9]; however, liver resection for patients with trans-glissonian involvement of the liver appears so effective that it brings survival close to the ones with IIIC stage ovarian cancer [12]. In the present study, an improved rate of survival for patients with peritoneal liver metastases was seen at the moment of primary, secondary, tertiary, and quaternary cytoreductions; however, these differences did not achieve statistical significance. The median survival of patients with hepatectomy for liver metastases from peritoneal seeding (*n* = 12) was higher than that of patients with hepatectomy for liver metastases from hematogenous origin (*n* = 19) (16.08 versus 12.66 months, log-rank *p* = 0.523) (Fig. 2). At primary cytoreduction, we noticed a difference of survival following hepatectomy regarding the mechanisms of liver involvement: 34.33 months survival following hepatectomy for patients with liver metastases from peritoneal seeding versus 15.63 months survival for the ones with hematogenous origin (log-rank *p* = 0.702). However, three patients with hepatectomy as part of primary cytoreductive surgery were alive at 139, 128, and 122 months of follow-up. A difference of survival regarding the mechanisms of involvement is maintained in the setting of secondary cytoreduction with an overall survival of 22 months for patients with liver metastases of peritoneal origin versus 10 months for the ones with hematogenous origin. Interestingly, patients submitted to hepatectomy during tertiary cytoreduction obtained impressive survival, with median survival of 62.86 months (range, 1–128).

In a study on 27 patients with liver resection at the time of secondary cytoreduction, Kolev et al. showed that the interval from the primary surgery (<24 months) and optimal secondary cytoreduction was significantly associated with the longest survival [10].

Liver resection remains safe with 0 mortality and a 25 % morbidity but with most complications solved in a conservatory manner.

## Conclusions

Liver resection for advanced ovarian cancer is a safe procedure for primary up to quaternary cytoreduction and may bring survival benefit. There is a difference in prognosis following hepatectomy between patients with liver metastases via hematogenous spread and the ones with liver involvement via peritoneal contamination in favor of the latter category.
